# Identification of Discriminatory Factors and Construction of a Nomogram for Differentiating IgG4‐RD and DLBCL

**DOI:** 10.1002/iid3.70529

**Published:** 2026-09-28

**Authors:** Yongkang Chen, Weimiao Li, Zhou Zhou, Jiqing Wu, Dongmei Zhou

**Affiliations:** ^1^ The First Clinical College Xuzhou Medical University Xuzhou China; ^2^ Rheumatology and Immunology Department The Affiliated Hospital of Xuzhou Medical University Xuzhou China

**Keywords:** differential diagnosis, diffuse large B‐cell lymphoma (DLBCL), IgG4‐related disease (IgG4‐RD), predictive nomogram, retrospective analysis

## Abstract

**Objective:**

To determine clinically useful indicators that distinguish IgG4‐related disease (IgG4‐RD) from diffuse large B‐cell lymphoma (DLBCL) and to construct a predictive model that may assist early identification.

**Methods:**

We retrospectively analyzed 19 patients with IgG4‐RD and 37 with DLBCL who presented with lymphadenopathy and received treatment at the Affiliated Hospital of Xuzhou Medical University between January 2012 and October 2025. The distribution of continuous variables was checked before group comparisons. Data with an approximately normal distribution are expressed as mean ± standard deviation (mean ± SD) and were compared by independent‐samples *t*‐tests; skewed data are reported as median (interquartile range [IQR]) and were compared by the Mann‐Whitney U test. Categorical variables are presented as counts and percentages (%) and were compared using the χ^2^ test or Fisher's exact test, as appropriate. Variables significant in univariate analyses were taken forward into multivariable logistic regression. Predictive performance was evaluated by receiver operating characteristic (ROC) analysis, calibration analysis, and decision curve analysis (DCA).

**Results:**

Multivariable logistic regression retained lymphocyte count, serum total protein, and prothrombin time (PT) as independent predictive factors. The model yielded an area under the receiver operating characteristic curve (AUC) of 0.91 (95% confidence interval [CI]: 0.82–0.99). Its calibration curve had a mean absolute error of 0.032. Across threshold probabilities from 0.2 to 1.0, decision curve analysis indicated greater net benefit than the “treat‐all” and “treat‐none” approaches.

**Conclusion:**

In this cohort, the predictive model showed potential to assist differentiation between IgG4‐RD and DLBCL. Because the study was retrospective, single‐center, and based on a limited sample, these findings should be regarded as preliminary and require confirmation in larger independent cohorts before clinical use.

**Abbreviations**
ACR: American College of Rheumatology
AGP: α1‐acid glycoprotein
ALP: alkaline phosphatase
ALT: alanine aminotransferase
APTT: activated partial thromboplastin time
AST: aspartate aminotransferase
AUC: the area under the receiver operating characteristic curve
A/G: albumin‐to‐globulin ratio
BMI: body mass index
CI: confidence interval
CK: creatine kinase
CK‐MB: creatine kinase isoenzyme
DCA: decision curve analysis
DLBCL: diffuse large B‐cell lymphoma
ESMO: European Society for Medical Oncology
EULAR: European League Against Rheumatism
FDP: fibrinogen degradation product
HB: hemoglobin
HL: Hodgkin lymphoma
hs‐cTnT: high‐sensitivity cardiac troponin T
IgG4‐RD: IgG4‐related disease
INR: international normalized ratio
IQR: interquartile range
LDH: lactate dehydrogenase
MAE: mean absolute error
MCH: mean corpuscular hemoglobin
MCHC: mean corpuscular hemoglobin concentration
MCV: mean corpuscular volume
MPV: mean platelet volume
NCCN: National Comprehensive Cancer Network
NHL: non‐Hodgkin lymphoma
OR: odds ratio
PDW: platelet distribution width
PLT: platelet count
PT: prothrombin time
RBC: red blood cell count
RDW‐SD: standard deviation of red cell distribution width
ROC: receiver operating characteristic
SD: standard deviation
Tfh: follicular helper T
Th2: T helper 2
WBC: white blood cell count

## 1 Introduction

IgG4‐related disease (IgG4‐RD) is a chronic fibroinflammatory condition driven by immune dysregulation [1]. Its etiology is not fully understood, and the clinical course is often indolent, with marked heterogeneity among patients. The disease can affect many organs and may produce tumor‐like enlargement of the lacrimal or major salivary glands, pancreas, lungs, lymph nodes, and other tissues, sometimes resembling malignant disease. Typical histological findings include an IgG4‐positive plasma‐cell^−^rich inflammatory infiltrate with T cells, B cells, and eosinophils, accompanied by storiform fibrosis and obliterative phlebitis [2]. No laboratory test alone establishes the diagnosis. Although serum IgG4 elevation is incorporated into current diagnostic frameworks, its diagnostic sensitivity and specificity remain limited [2, 3]. Therefore, IgG4‐RD is diagnosed by integrating clinical, radiological, laboratory, and histopathological evidence [4]. Its combined inflammatory and fibrotic nature helps explain the broad variation in manifestations across organs and individuals.

Lymphoma is a clonal malignancy of the lymphohematopoietic system arising from genetic abnormalities acquired during the differentiation and maturation of T, B, or NK lymphocytes, with subsequent expansion of the affected cell population (e.g., CD4^+^ T cells or CD19^+^ B cells) [5]. Clinically, lymphomas are broadly separated into Hodgkin lymphoma (HL) and non‐Hodgkin lymphoma (NHL) [6]; NHL is further classified by lineage into B‐cell, T‐cell, and NK/T‐cell neoplasms [7]. B‐cell NHL represents about 85–90% of NHL, and diffuse large B‐cell lymphoma (DLBCL) is the predominant subtype, accounting for approximately 25–50% of newly diagnosed NHL cases each year [8]. DLBCL may present with rapidly enlarging lymph nodes, B symptoms, fatigue, hepatosplenomegaly, or involvement of other organs. Around 40% of patients have extranodal disease, commonly affecting sites such as the gastrointestinal tract, bone, central nervous system, or skin [9]. National Cancer Center data from China reported an overall 5‐year survival of about 38.3% for lymphoma in 2018 [10]. Given the aggressive nature of DLBCL, early recognition is especially important when lymphadenopathy is the main initial finding.

Overlap in the clinical presentation of IgG4‐RD and DLBCL can make early differentiation difficult. Histopathological biopsy is frequently needed for confirmation, which can prolong the diagnostic process and add to the burden of evaluation. Distinguishing the two disorders promptly is clinically important because their treatment and prognosis differ substantially. To date, publications addressing both diseases have mainly described their coexistence or sequential development [11, 12, 13, 14], and systematic case‐control comparisons designed to identify discriminatory features or create a clinical decision tool remain scarce. In this retrospective study, we therefore sought to identify routinely available indicators that may separate IgG4‐RD from DLBCL and to develop a non‐invasive prediction model for early assessment. Such an approach could provide supplementary guidance while definitive pathological evaluation is pending.

## 2 Materials and Methods

### 2.1 Study Population

We retrospectively identified 19 newly diagnosed patients with IgG4‐RD and 37 newly diagnosed patients with DLBCL who presented with lymphadenopathy and received care at the Affiliated Hospital of Xuzhou Medical University between January 2012 and October 2025.

Patients were eligible for the IgG4‐RD group if they: (1) were aged ≥ 18 years; (2) satisfied either the 2019 ACR/EULAR classification criteria [4] or the 2011 Japanese comprehensive diagnostic criteria for IgG4‐RD [15]; (3) had not received previous systemic treatment for IgG4‐RD; and (4) had lymphadenopathy at the initial presentation. The two diagnostic/classification frameworks and their source references are summarized in Tables S1 and S2.

Eligibility for the DLBCL group required: (1) age ≥ 18 years; (2) tissue‐based confirmation of DLBCL by experienced hematopathologists according to the applicable WHO classification of haematolymphoid tumors; (3) no previous systemic therapy for DLBCL; and (4) lymphadenopathy at presentation. All 37 DLBCL cases underwent routine immunohistochemical assessment. In routine clinical practice, fluorescence in situ hybridization (FISH) was additionally performed in 8 patients with suspected high‐grade disease or other high‐risk clinicopathological features. Available FISH results did not support a final diagnosis of double‐hit or triple‐hit lymphoma in any of these eight patients. This additional molecular evaluation was used only when clinically indicated and did not alter the predefined cohort definition.

Patients with any concomitant malignancy were excluded from the IgG4‐RD group, whereas those with malignancies other than DLBCL were excluded from the DLBCL group. For both groups, we also excluded patients with other immune‐mediated diseases, including Sjögren's disease, adult‐onset Still's disease, hemophagocytic syndrome, and autoimmune liver disease, as well as those with a recent acute bacterial infection such as an upper respiratory tract infection or sepsis.

Within the first 24 h after admission, 62 variables were extracted, including demographic characteristics (age, sex, body mass index [BMI], and disease duration) and laboratory measurements such as complete blood count, biochemical indices, and selected immunological tests. These variables were subsequently compared between the two diagnostic groups.

For sensitivity analysis, we formed a subgroup of biopsy‐confirmed IgG4‐RD cases and age‐ and sex‐matched DLBCL cases. Model development was based on the complete cohort (*n* = 56); the matched biopsy‐proven subset (12 patients in each group) was used only to explore the possible effect of diagnostic misclassification and was not used to build, refit, or independently validate the model. Because IgG4‐RD is uncommon and the study was retrospective, every consecutive patient who fulfilled the predefined eligibility criteria between January 2012 and October 2025 was included. This design was chosen to preserve the maximum available sample while separately examining whether diagnostic certainty altered the main pattern of results.

Ethical approval was obtained from the Ethics Committee of the Affiliated Hospital of Xuzhou Medical University (No. XYFY2025‐KL206‐01). The study followed the Declaration of Helsinki and applicable local requirements, and written informed consent was obtained from all participants.

### 2.2 Statistical Analysis

All analyses were conducted with SPSS version 27.0 (IBM Corp., Armonk, NY, USA). Distributional normality of continuous variables was evaluated with the Shapiro–Wilk test. Data meeting normality assumptions are expressed as mean ± standard deviation (mean ± SD) and were compared between groups by an independent‐samples *t*‐test. Variables with non‐normal distributions are summarized as median (interquartile range [IQR]) and were assessed with the Mann‐Whitney U test. Categorical variables are reported as number and percentage (%) and were compared using the χ^2^ test or Fisher's exact test when appropriate. No imputation was performed for missing observations.

For biopsy‐proven cases with available tissue, immunohistochemical information was collected for CD20, CD19, PAX5, CD138, MUM1, CD38, CD3, CD5, CD10, BCL6, EBER, and Ki‐67. To examine possible diagnostic misclassification, the biopsy‐proven subgroup was analyzed after 1:1 greedy nearest‐neighbor matching on sex and age within ± 10 years. Comparisons of baseline characteristics in the matched groups followed the same statistical procedures described above. Immunohistochemical findings were used only in this sensitivity analysis and were not incorporated into the primary prediction model.

Variables significant in univariate testing were subsequently included in the multivariable logistic regression model. We characterized model discrimination using receiver operating characteristic (ROC) curves, assessed agreement between predicted and observed probabilities with calibration curves, and explored potential clinical usefulness by decision curve analysis (DCA). All tests were two‐sided, with *p* < 0.05 considered statistically significant.

## 3 Results

### 3.1 Baseline Demographic Data and Laboratory Indicators

The final cohort contained 56 patients, including 19 with IgG4‐RD and 37 with DLBCL. Between‐group comparisons identified 20 variables with *p* < 0.05: disease duration, white blood cell count (WBC), neutrophil percentage, eosinophil percentage, lymphocyte count, eosinophil count, basophil count, mean corpuscular volume (MCV), mean corpuscular hemoglobin (MCH), prothrombin time (PT), international normalized ratio (INR), serum total protein, serum globulin, albumin‐to‐globulin ratio (A/G), creatinine, Na^+^, HCO_3_^−^, lactate dehydrogenase (LDH), complement C3, and complement C4 (Table 1). These comparisons were exploratory and served to identify variables for subsequent regression analyses rather than to establish disease‐specific biomarkers.

**Table 1:** Baseline demographic data and laboratory indicators between IgG4‐RD group and DLBCL group.

Variables	DLBCL group (*n* = 37)		IgG4‐RD group (*n* = 19)		*p*
	mean/median	SD/IQR	mean/median	SD/IQR	
Sex	—	—	—	—	0.645
Female	16	—	7	—	—
Man	21	—	12	—	—
Age(years)	59.38	16.12	57.11	14.15	0.605
BMI (kg/m^2^)	24.56	(22.00, 28.56)	22.58	(21.97, 24.39)	0.097
disease duration (month)	1.00	(0.65, 2.50)	3.00	(1.00, 12.00)	0.003
WBC (×10^9^/L)	5.6	(4.40, 6.81)	6.1	(5.50, 8.10)	0.029
RBC (×10^12^/L)	4.30	0.76	4.04	0.79	0.243
PLT (×10^9^/L)	229.00	(184.50, 277.00)	225.00	(153.00, 273.00)	0.406
Neutrophil percentage (%)	66.92	12.52	58.66	14.76	0.032
Lymphocyte percentage (%)	20.20	(13.70, 30.00)	24.00	(21.70, 33.50)	0.166
Monocyte percentage (%)	8.09	3.23	6.55	2.03	0.065
Eosinophil percentage (%)	1.40	(0.65, 2.70)	2.30	(1.30, 7.20)	0.046
Basophil percentage (%)	0.40	(0.30, 0.65)	0.60	(0.30, 0.70)	0.243
Lymphocyte count (×10^9^/L)	1.17	0.60	1.80	0.57	< 0.001
Neutrophils count (×10^9^/L)	3.40	(2.35, 5.32)	4.13	(2.93, 4.77)	0.387
Monocyte count (×10^9^/L)	0.40	(0.32, 0.52)	0.44	(0.32, 0.53)	0.742
Eosinophils count (×10^9^/L)	0.08	(0.04, 0.12)	0.15	(0.04, 0.43)	0.023
Basophils count (×10^9^/L)	0.02	(0.01, 0.03)	0.04	(0.02, 0.05)	0.014
HB (g/L)	134.00	(105.50, 144.00)	125.00	(112.00, 135.00)	0.522
Hematocrit (%)	39.40	(33.40, 44.60)	39.60	(34.90, 42.50)	0.665
MCV (fL)	90.70	(86.85, 94.25)	93.40	(91.50, 97.10)	0.008
MCH (pg)	29.20	(28.25, 30.85)	30.40	(29.20, 32.20)	0.021
MCHC (g/L)	326.00	(316.00, 333.50)	327.00	(319.00, 332.00)	0.869
RDW‐SD (fL)	42.40	(39.80, 45.25)	44.40	(41.10, 47.60)	0.134
RDW‐CV (%)	12.70	(12.25, 14.35)	13.30	(12.30, 14.10)	0.556
PDW (fL)	14.80	(11.35, 15.95)	11.80	(10.60, 15.80)	0.451
MPV (fL)	10.14	1.11	10.53	1.41	0.270
APTT (s)	27.30	(25.15, 28.80)	27.70	(26.20, 29.20)	0.610
PT (s)	10.91	0.86	11.61	1.35	0.024
INR	0.94	0.07	1.00	0.11	0.023
Fibrinogen (g/L)	2.91	(2.47, 4.49)	3.04	(2.41, 4.14)	0.622
D‐dimer (µg/mL)	0.53	(0.19, 1.06)	0.34	(0.20, 2.12)	0.951
FDP (µg/mL)	2.75	(1.65, 3.92)	2.00	(1.20, 6.30)	0.549
AST (U/L)	19.00	(15.00, 24.00)	22.00	(16.00, 34.00)	0.171
ALT (U/L)	18.00	(11.00, 25.50)	19.00	(16.00, 35.00)	0.209
ALP (U/L)	77.00	(62.50, 100.00)	76.00	(61.00, 97.00)	0.775
Prealbumin (g/L)	0.240	(0.138, 0.285)	0.230	(0.130, 0.275)	0.543
Serum total protein (g/L)	67.21	7.18	79.87	12.68	< 0.001
Serum albumin (g/L)	41.20	(37.5, 44.90)	40.50	(31.10, 42.60)	0.276
Serum globulin (g/L)	26.95	(22.5, 31.02)	36.20	(28.90, 55.50)	< 0.001
A/G	1.55	0.41	1.07	0.49	< 0.001
Total bilirubin (μmol/L)	10.80	(7.85, 14.75)	9.50	(7.20, 16.30)	0.947
Direct bilirubin (μmol/L)	4.10	(2.85, 5.55)	3.50	(2.90, 4.60)	0.505
Total bile acids (μmol/L)	4.10	(2.35, 7.35)	5.10	(3.00, 9.95)	0.361
Cholinesterase (U/L)	7783	(5283.5, 8758.5)	7088	(4971, 8238)	0.272
Urea (mmol/L)	4.89	(3.9, 5.98)	5.92	(4.31, 7.07)	0.114
Creatinine (μmol/L)	58.00	(42.00, 66.50)	69.50	(54.00, 93.75)	0.008
Uric acid (μmol/L)	301.00	(233.50, 343.00)	323.50	(255.50, 410.25)	0.307
K^+^ (mmol/L)	4.11	0.30	4.15	0.29	0.625
Na^+^ (mmol/L)	140.60	(138.70, 141.65)	138.65	(136.07, 140.07)	0.014
Cl^‐^ (mmol/L)	102.19	2.88	102.74	3.26	0.526
Mg^2+^ (mmol/L)	0.88	(0.83, 0.92)	0.86	(0.84, 0.93)	0.898
Ca^2+^ (mmol/L)	2.26	0.13	2.23	0.13	0.402
HCO_3_^−^(mmol/L)	23.80	(22.55, 25.60)	22.90	(21.50, 23.95)	0.047
LDH (U/L)	200.00	(177.00, 303.50)	172	(157.75, 200.50)	0.012
CK (U/L)	48	(29.2, 71.50)	59.50	(28.2, 89.00)	0.430
CK‐MB (U/L)	0.97	(0.52, 1.33)	1.24	(0.89, 1.57)	0.116
hs‐cTnT (ng/L)	5.56	(4.1, 8.22)	4.68	(3.05, 7.38)	0.347
AGP (g/L)	80.00	(63.7, 118.10)	69.80	(60.0, 87.10)	0.181
IgA (g/L)	1.97	(1.47, 2.76)	1.53	(1.01, 2.29)	0.161
IgM (g/L)	0.81	(0.55, 1.10)	0.71	(0.46, 1.01)	0.505
Complement C3 (g/L)	1.05	0.20	0.65	0.33	< 0.001
Complement C4 (g/L)	0.26	0.06	0.14	0.10	< 0.001

*Note:* All variables were collected at baseline. Variables showing *p* < 0.05 in the baseline comparisons were evaluated in univariate logistic regression.

Abbreviations: A/G, albumin‐to‐globulin ratio; AGP, α1‐acid glycoprotein; ALP, alkaline phosphatase; ALT, alanine aminotransferase; APTT, activated partial thromboplastin time; AST, aspartate aminotransferase; CK, creatine kinase; CK‐MB, creatine kinase isoenzyme; FDP, fibrinogen degradation products; HB, hemoglobin; INR, international normalized ratio; LDH, lactate dehydrogenase; MCH, mean corpuscular hemoglobin; MCHC, mean corpuscular hemoglobin concentration; MCV, mean corpuscular volume; MPV, mean platelet volume; PDW, platelet distribution width; PLT, platelet count; PT, prothrombin time; RBC, red blood cell count; RDW‐SD, standard deviation of red cell distribution width; T = hs‐cTnT, high‐sensitivity cardiac troponin; WBC, white blood cell count.

### 3.2 Sensitivity Analysis in the Matched Biopsy‐Proven Subgroup

To explore whether diagnostic misclassification influenced the results, we restricted an additional analysis to pathologically confirmed patients and performed 1:1 matching by age and sex. This procedure yielded 12 matched pairs (24 patients). Age (*p* = 0.754) and sex (*p* = 1.000) were comparable between the two matched groups (Table 2).

**Table 2:** Baseline characteristics of the matched biopsy‐proven subgroup used for sensitivity analysis.

Variables	DLBCL group (*n* = 12)		IgG4‐RD group (*n* = 12)		*P*
	mean/median	SD/IQR	mean/median	SD/IQR	
Sex	—	—	—	—	1.000
Female	3	—	4	—	—
Man	9	—	8	—	—
Age (years)	55.17	9.18	53.75	12.45	0.754
BMI (kg/m^2^)	24.91	(24.48, 27.03)	22.83	(22.03, 23.88)	0.030
Disease duration (month)	1.00	(0.50, 1.00)	5.50	(1.75, 12.00)	0.001
WBC (×10^9^/L)	5.70	(4.80, 6.53)	6.10	(5.47, 7.30)	0.298
RBC (×10^12^/L)	4.58	0.58	4.36	0.61	0.368
PLT (×10^9^/L)	223.83	33.22	211.50	49.45	0.481
Neutrophil percentage (%)	64.42	12.65	55.95	12.36	0.111
Lymphocyte percentage (%)	24.45	(19.93, 28.10)	24.80	(22.97, 30.60)	0.514
Monocyte percentage (%)	7.12	2.99	6.67	2.01	0.670
Eosinophil percentage (%)	1.20	(0.70, 3.25)	3.60	(1.85, 8.72)	0.030
Basophil percentage (%)	0.35	(0.30, 0.53)	0.70	(0.47, 0.85)	0.085
Lymphocyte count (×10^9^/L)	1.45	(1.17, 1.63)	1.75	(1.40, 1.92)	0.099
Neutrophils count (×10^9^/L)	4.04	1.75	3.70	1.04	0.570
Monocyte count (×10^9^/L)	0.43	0.19	0.43	0.13	0.902
Eosinophils count (×10^9^/L)	0.08	(0.04, 0.18)	0.19	(0.19, 0.66)	0.030
Basophils count (×10^9^/L)	0.02	(0.02, 0.03)	0.04	(0.03, 0.07)	0.050
HB (g/L)	135.33	23.85	132.50	18.90	0.750
Hematocrit (%)	41.57	5.67	40.47	5.23	0.626
MCV (fL)	92.90	(88.25, 95.60)	92.85	(91.65, 94.35)	0.750
MCH (pg)	30.60	(28.80, 32.00)	30.30	(29.85, 31.04)	1.000
MCHC (g/L)	330.00	(318.00, 338.75)	328.00	(320.75, 332.75)	0.795
RDW‐SD (fL)	42.30	(40.68, 46.58)	43.00	(41.08, 45.27)	0.817
RDW‐CV (%)	13.05	(12.40, 15.43)	12.95	(12.28, 13.72)	0.686
PDW (fL)	15.90	(14.65, 16.22)	12.70	(10.85, 15.57)	0.140
MPV (fL)	10.00	1.36	10.43	1.27	0.437
APTT (s)	27.40	(24.50, 28.65)	27.75	(26.48, 30.35)	0.453
PT (s)	10.35	(10.00, 10.90)	11.25	(10.95, 11.70)	0.030
INR	0.90	(0.86, 0.95)	0.97	(0.95, 1.02)	0.032
Fibrinogen (g/L)	2.53	(2.21, 3.77)	2.67	(2.30, 3.11)	0.453
d‐dimer (µg/mL)	0.29	(0.18, 0.44)	0.24	(0.20, 0.30)	0.906
FDP (µg/mL)	2.40	(1.40, 2.60)	1.80	(1.52, 2.38)	0.953
AST (U/L)	18.00	(15.75, 21.25)	17.50	(14.00, 25.50)	0.862
ALT (U/L)	21.50	(12.00, 37.25)	17.00	(12.50, 20.75)	0.602
ALP (U/L)	72.50	(59.50, 90.50)	70.50	(60.50, 78.50)	0.729
Prealbumin (g/L)	0.27	0.09	0.22	0.07	0.181
Serum total protein (g/L)	72.15	(67.77, 73.55)	77.25	(70.70, 83.93)	0.052
Serum albumin (g/L)	43.35	(40.10, 45.55)	41.10	(35.40, 44.23)	0.419
Serum globulin (g/L)	27.95	(26.40, 31.38)	33.35	(28.25, 48.45)	0.100
A/G	1.49	0.37	1.16	0.57	0.105
Total bilirubin (μmol/L)	10.75	(8.20, 15.05)	10.05	(8.85, 13.85)	0.862
Direct bilirubin (μmol/L)	4.36	1.52	3.76	1.59	0.355
Total bile acids (μmol/L)	3.90	(2.30, 5.60)	3.00	(2.70, 6.40)	0.818
Cholinesterase (U/L)	8758.50	(7930.25, 8942.00)	7492.50	(5412.00, 8302.25)	0.089
Urea (mmol/L)	4.54	(3.72, 5.33)	5.92	(5.44, 6.63)	0.002
creatinine (μmol/L)	61.00	(55.00, 66.75)	74.00	(59.25, 87.25)	0.083
Uric acid (μmol/L)	337.00	(267.25, 369.00)	355.00	(281.25, 374.55)	0.729
K^+^ (mmol/L)	3.98	0.31	4.17	0.24	0.118
Na^+^ (mmol/L)	139.60	(139.23, 140.90)	139.00	(137.50, 139.62)	0.165
Cl^‐^ (mmol/L)	101.45	3.16	102.85	3.78	0.335
Mg^2+^ (mmol/L)	0.90	(0.82, 0.95)	0.87	(0.86, 0.94)	0.817
Ca^2+^ (mmol/L)	2.28	0.12	2.22	0.14	0.272
HCO_3_^−^ (mmol/L)	23.67	2.28	22.69	2.96	0.383
LDH (U/L)	204.00	(175.50, 281.50)	172.00	(164.50, 190.75)	0.049
CK (U/L)	62.00	(36.75, 70.50)	60.50	(48.50, 89.00)	0.713
CK‐MB (U/L)	0.94	(0.56, 1.40)	1.24	(0.95, 1.50)	0.193
hs‐cTnT (ng/L)	6.67	(4.07, 7.88)	4.61	(3.28, 5.82)	0.459
AGP (g/L)	69.50	11.52	62.29	13.26	0.253
IgA (g/L)	1.85	(1.42, 2.66)	1.52	(1.25, 2.23)	0.378
IgM (g/L)	0.86	(0.65, 1.00)	0.68	(0.47, 0.99)	0.378
Complement C3 (g/L)	1.08	0.18	0.68	0.31	0.002
Complement C4 (g/L)	0.26	0.05	0.14	0.10	0.004
EBER	—	—	—	—	0.214
0	11	2	11	2	—
1	0	1	0	1	—
CD138	—	—	—	—	0.143
0	3	0	3	0	—
1	1	3	1	3	—
MUM1	—	—	—	—	0.423
0	1	1	1	1	—
1	9	2	9	2	—
CD38	—	—	—	—	0.143
0	3	0	3	0	—
1	1	4	1	4	—
CD3	—	—	—	—	0.102
0	5	0	5	0	—
1	6	6	6	6	—
CD5	—	—	—	—	0.234
0	6	0	6	0	—
1	6	4	6	4	—
CD10	—	—	—	—	0.015
0	8	0	8	0	—
1	2	4	2	4	—

*Note:* For immunohistochemical variables, results are shown as numbers of patients according to expression status (0 = negative; 1 = positive). Immunohistochemical information was not available for every patient.

Several differences observed in the complete cohort were also present after restriction to the matched biopsy‐proven subgroup. PT, complement C3, complement C4, BMI, disease duration, eosinophil percentage, eosinophil count, INR, urea, LDH, and CD10 remained significantly different between the IgG4‐RD and DLBCL groups (all *p* < 0.05). Because immunohistochemical results were available for only a small number of specimens, the CD10 finding should be viewed as exploratory. Neutrophil percentage, lymphocyte count, basophil count, MCV, MCH, serum total protein, serum globulin, A/G, Na^+^, and HCO_3_^‐^ changed in directions similar to those in the full cohort but no longer reached statistical significance, probably reflecting the reduced sample size (*n* = 24 vs. *n* = 56). Thus, some full^−^cohort group differences were reproduced in the biopsy‐proven subset, although the small subgroup means that this analysis is exploratory and cannot be regarded as independent validation of the nomogram. The loss of significance for several variables in this subset should therefore not be interpreted as evidence that the corresponding full‐cohort differences were absent.

### 3.3 Univariate and Multivariable Logistic Regression Analysis

Both univariate and multivariable logistic regression analyses used the complete cohort (*n* = 56; 19 IgG4‐RD and 37 DLBCL). Five key baseline variables were examined in univariate models: lymphocyte count, serum total protein, PT, eosinophil percentage, and HCO_3_^−^. Each showed statistical significance: lymphocyte count (*p* = 0.003, odds ratio [OR] = 5.69, 95% confidence interval [CI]: 1.84–17.61), serum total protein (*p* = 0.001, OR = 1.17, 95% CI: 1.06–1.29), PT (*p* = 0.037, OR = 1.85, 95% CI: 1.04–3.31), eosinophil percentage (*p* = 0.042, OR = 1.34, 95% CI: 1.01–1.78), and HCO_3_^−^ (*p* = 0.032, OR = 0.74, 95% CI: 0.57–0.97). These variables were then assessed together in a multivariable logistic regression model. Lymphocyte count (*p* = 0.032, OR = 12.20, 95% CI: 1.24–119.69), serum total protein (*p* = 0.026, OR = 1.21, 95% CI: 1.02–1.43), and PT (*p* = 0.049, OR = 4.16, 95% CI: 1.01–17.21) remained independently associated with group classification (Table 3). The multivariable model was used to determine which of the candidate variables retained an independent association after simultaneous adjustment for the other included factors.

**Table 3:** Univariate and multivariable logistic regression of key baseline parameters.

Factors	Univariate analysis		Multivariable analysis	
	*p*	OR (95% CI)	*p*	OR (95% CI)
Lymphocyte count (×10^9^/L)	0.003	5.69 (1.84 ~ 17.61)	0.032	12.20 (1.24 ~ 119.69)
Serum total protein (g/L)	0.001	1.17 (1.06 ~ 1.29)	0.026	1.21 (1.02 ~ 1.43)
PT (s)	0.037	1.85 (1.04 ~ 3.31)	0.049	4.16 (1.01 ~ 17.21)
Eosinophil percentage (%)	0.042	1.34 (1.01 ~ 1.78)	—	—
HCO_3_^−^ (mmol/L)	0.032	0.74 (0.57 ~ 0.97)	—	—

Abbreviation: PT, prothrombin time.

### 3.4 Development and Performance Assessment of the Nomogram

The nomogram incorporated the three variables that remained in the multivariable model: lymphocyte count, serum total protein, and PT (Figure 1). ROC analysis showed an AUC of 0.91 (95% CI: 0.82–0.99) (Table 4, Figure 2). The calibration analysis produced a mean absolute error of 0.032 (Figure 3). In DCA, the model provided a potential net benefit relative to the “treat‐all” and “treat‐none” strategies over threshold probabilities of 0.2–1.0 (Figure 4). These analyses describe internal model performance in the present cohort and should not be interpreted as external validation.

**Figure 1:**
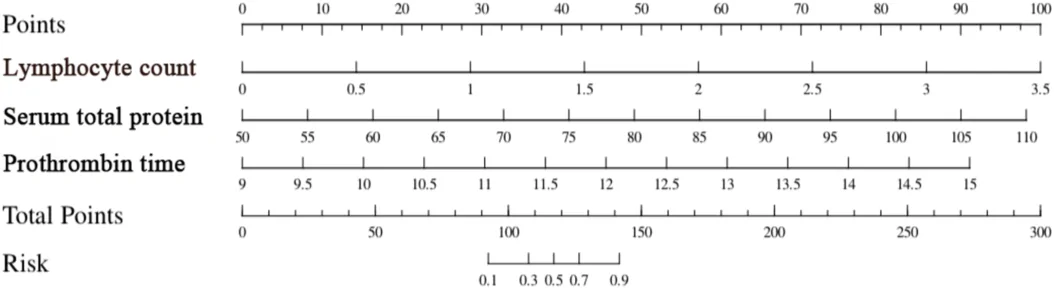
Nomogram for the predictive model.

**Table 4:** Receiver operating characteristic (ROC) curve for the predictive model.

AUC (95% CI)	Accuracy (95% CI)	Sensitivity (95% CI)	Specificity (95% CI)	PPV (95% CI)	NPV (95% CI)	Cut off
0.91 (0.82–0.99)	0.88 (0.76–0.95)	0.84 (0.68−1.00)	0.89 (0.79−0.99)	0.80 (0.62−0.98)	0.92 (0.83−1.00)	0.354

**Figure 2:**
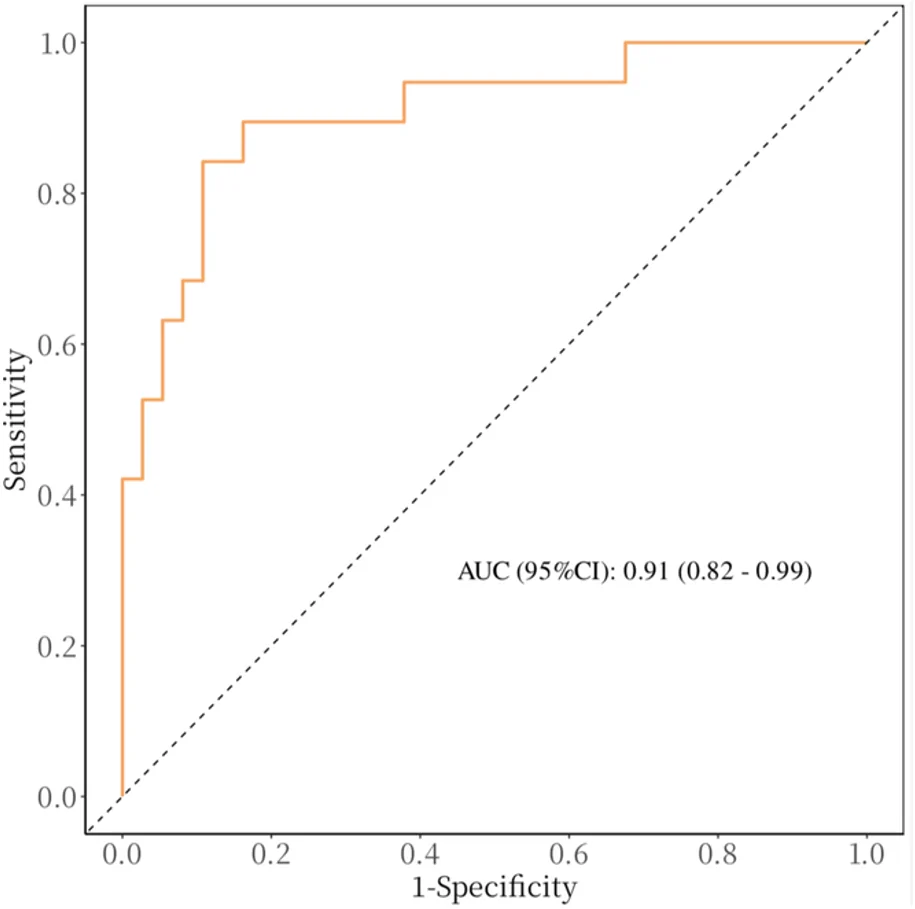
Receiver operating characteristic (ROC) curve for the predictive model.

**Figure 3:**
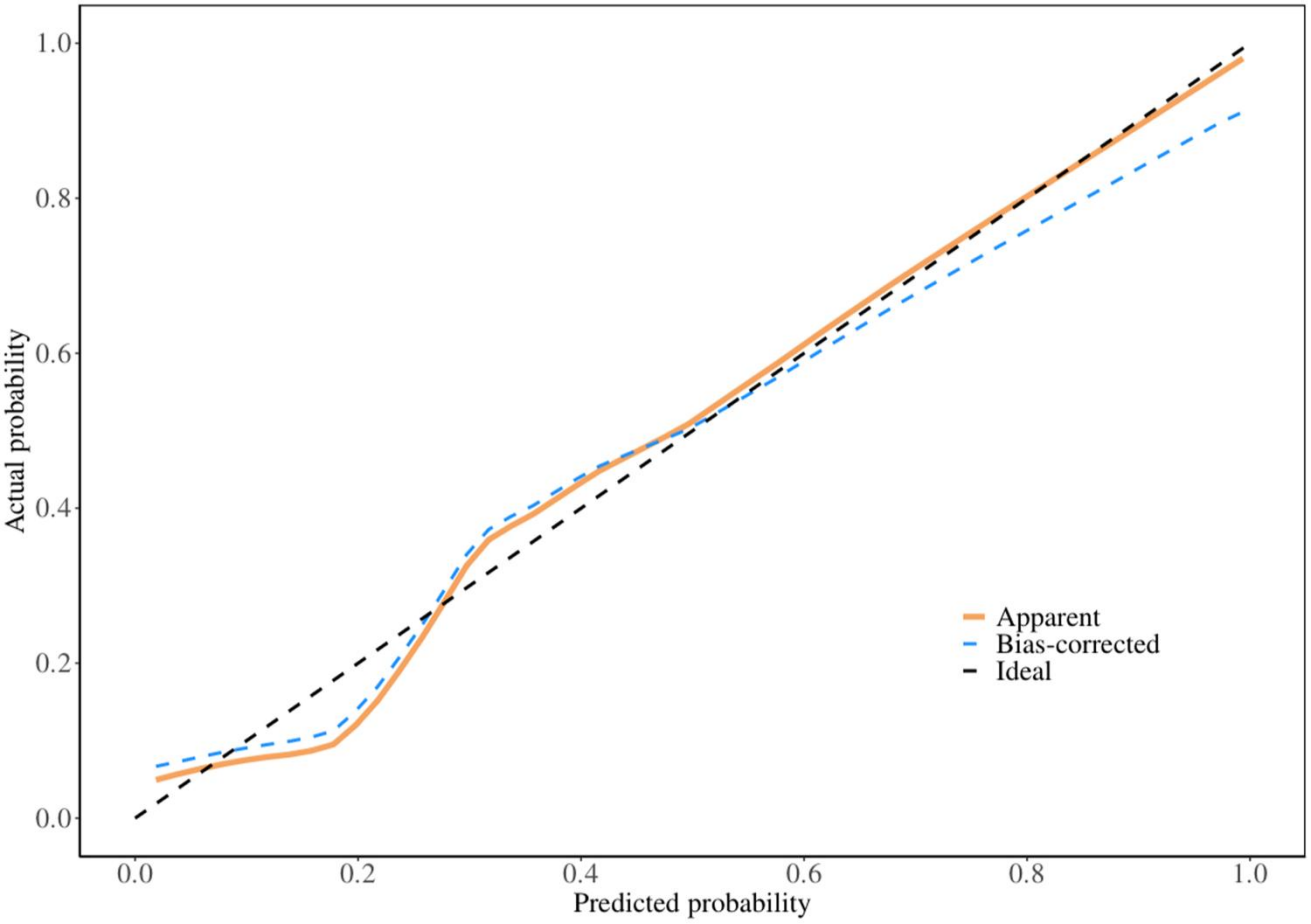
Calibration curve for the predictive model.

**Figure 4:**
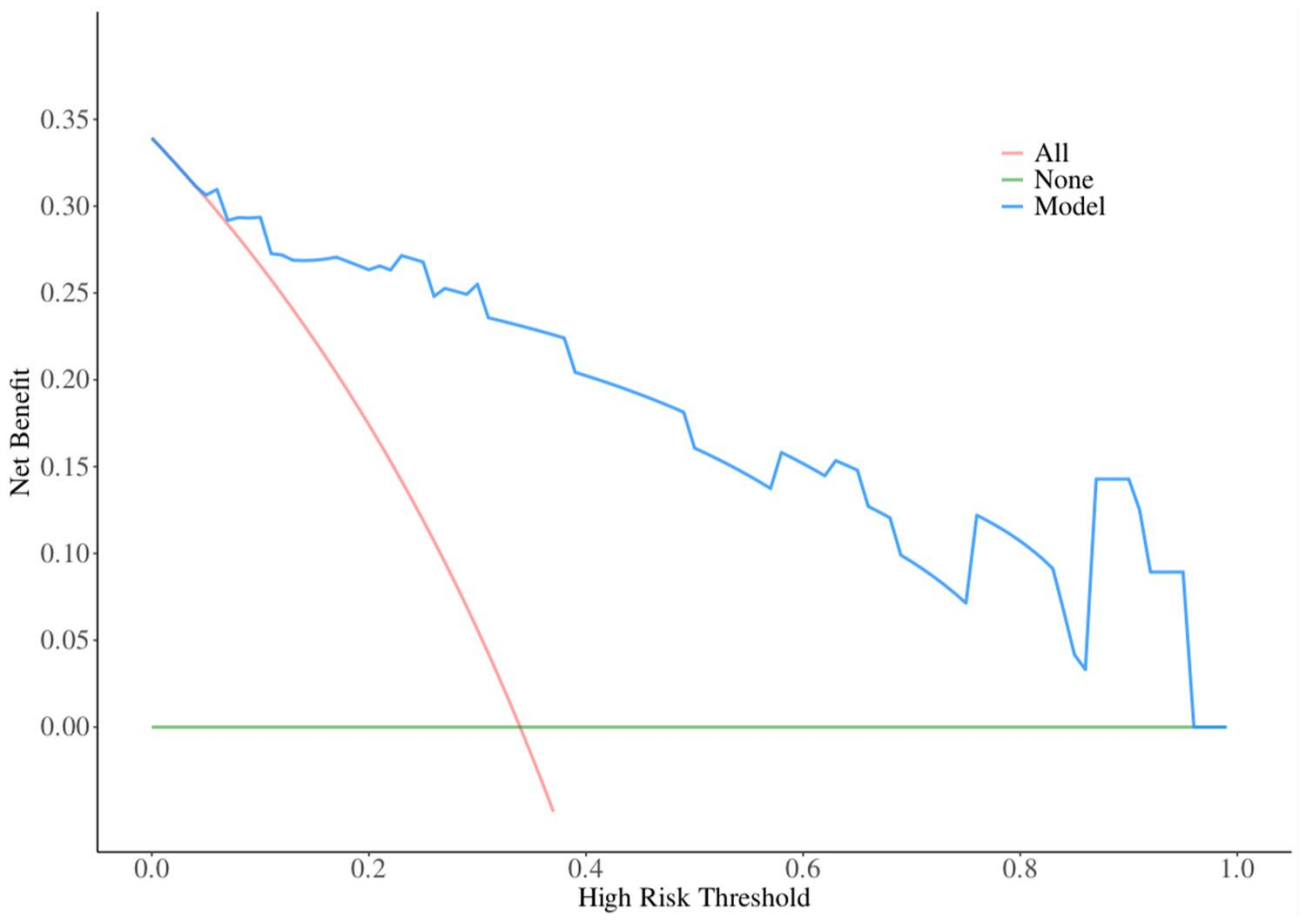
Decision curve analysis for the predictive model.

### 3.5 Post‐Hoc Power Analysis

Among the three independent predictors obtained from the full cohort—lymphocyte count, serum total protein, and PT—PT was the only variable that remained statistically significant in the matched biopsy‐proven subgroup (*p* = 0.030); the other two retained similar directional trends but were no longer significant (*p* = 0.099 and *p* = 0.052, respectively). We therefore used PT to define the effect size for a post‐hoc power calculation. With the observed PT values of 11.61 ± 1.35 versus 10.91 ± 0.86 s, sample sizes of 19 and 37, and a two‐sided alpha of 0.05, the estimated power was 53.8%. This relatively low power illustrates the statistical limitations imposed by the modest cohort size and supports interpreting the study as exploratory.

## 4 Discussion

Painless lymphadenopathy can be an initial manifestation of both IgG4‐RD and DLBCL, yet management of the two disorders is fundamentally different. IgG4‐RD is an autoimmune fibroinflammatory condition that generally responds to glucocorticoids, whereas treating presumed lymphoma may involve chemotherapy, radiotherapy, or surgery. If IgG4‐RD is mistaken for lymphoma, patients may therefore undergo unnecessary and potentially harmful treatment, including organ resection, which has been reported in this setting [16]. Conversely, failure to recognize DLBCL promptly may delay effective therapy. A definitive diagnosis of IgG4‐RD usually requires integration of laboratory, imaging, and pathological information, and this process can lengthen hospitalization and increase financial as well as physical and psychological burdens. These considerations support the need for a rapid, non‐invasive adjunct to aid early differential diagnosis.

IgG4‐RD and lymphoma have a complicated clinical relationship: they may arise independently, coexist, or appear at different times in the same patient. Lymphoma has been reported as the most frequent malignancy occurring in association with IgG4‐RD [17]. Although IgG4‐RD has at times been discussed as a possible premalignant state or paraneoplastic phenomenon, supporting evidence is inconclusive, and other studies do not support these interpretations; it is currently regarded primarily as an autoimmune disorder [18]. Irrespective of this pathogenic debate, the practical problem addressed here is the prompt distinction of IgG4‐RD from DLBCL. Previous studies have generally described the clinical features and outcomes of the two diseases separately [19, 20, 21], while reports involving both have mainly been individual cases [11, 12, 13, 14]. We therefore compared 19 patients newly diagnosed with IgG4‐RD from January 2012 to October 2025 with contemporaneous DLBCL patients presenting with lymphadenopathy and used univariate followed by multivariable logistic regression to derive a prediction model. The resulting factors may provide additional information for early differential diagnosis and timely selection of appropriate treatment. This study was therefore designed around a focused clinical question rather than an attempt to resolve the broader biological relationship between autoimmunity and lymphomagenesis. By emphasizing patients who presented with lymphadenopathy, we sought to evaluate a setting in which the two disorders can be particularly difficult to distinguish at initial assessment. The resulting model is intended to provide supplementary information while definitive diagnostic work‐up proceeds, rather than to substitute for established diagnostic procedures.

Twenty baseline variables differed significantly between groups (*p* < 0.05): disease duration, WBC, neutrophil percentage, eosinophil percentage, lymphocyte count, eosinophil count, basophil count, MCV, MCH, PT, INR, serum total protein, serum globulin, A/G, creatinine, Na^+^, HCO_3_^−^, LDH, complement C3, and complement C4. Five candidate variables—lymphocyte count, serum total protein, PT, eosinophil percentage, and HCO_3_^−^—were examined by univariate and then multivariable logistic regression. Lymphocyte count, serum total protein, and PT remained independently associated with the distinction between IgG4‐RD and DLBCL.

Lymphocyte count is a routinely available measure related to systemic immune status. In our cohort, peripheral lymphocyte counts were lower in DLBCL than in IgG4‐RD (1.17 ± 0.60 vs. 1.80 ± 0.57), consistent with previous observations [22]. This peripheral immunosuppressive pattern in DLBCL differs from the biology of its tumor microenvironment. Higher infiltration by T cells, non‐malignant B cells, and NK cells within lymphoma tissue has been associated with improved survival [23, 24], suggesting that local antitumour immune activity can remain prognostically relevant despite systemic immune suppression. IgG4‐RD, by contrast, is characterized by chronic immune activation. Peripheral immune profiling has demonstrated expansion of CD4^+^ T‐cell populations, including T helper 2 (Th2) and follicular helper T (Tfh) subsets [25, 26], in keeping with the prominent lymphoplasmacytic infiltration seen in affected tissues [27]. The observed difference in lymphocyte count may therefore reflect contrasting systemic immune states in DLBCL and IgG4‐RD and supports its use as a candidate discriminatory variable. This interpretation is consistent with the view that a simple peripheral blood measure may indirectly reflect broader differences in immune regulation between the two disorders. Nevertheless, lymphocyte count is influenced by many clinical factors and should not be considered specific for either disease when used in isolation.

Serum total protein provides a broad measure of circulating proteins, including immunoglobulins. Levels were higher in the IgG4‐RD group than in patients with DLBCL in our study. This difference is biologically plausible because IgG4‐RD frequently features hypergammaglobulinemia related to B‐cell activation and expansion of circulating plasmablasts [28]. Proteomic studies have also reported increases not only in IgG4 but in other immunoglobulin subclasses, including IgG1 and IgG2, as well as acute‐phase proteins such as alpha‐1‐antitrypsin [29]. Such polyclonal immunoglobulin production can raise the measured total protein concentration. DLBCL, in contrast, is associated with tumor‐related alterations of the serum proteome [30], while increased catabolism and impaired nutritional status may contribute to normal or reduced total protein concentrations in some patients. Thus, serum total protein may capture differences between sustained polyclonal immune activation in IgG4‐RD and the metabolic and proteomic disturbances associated with DLBCL. In addition to immunoglobulin production, differences in systemic inflammation, nutritional state, and tumor‐associated metabolism may contribute to the separation observed between groups. Accordingly, total protein is best interpreted as an integrated routine laboratory signal rather than as a disease‐specific marker. Its clinical value in the model may arise from capturing several of these biological processes simultaneously.

PT is a commonly used coagulation measurement, usually interpreted together with the INR for assessment of the extrinsic coagulation pathway and for anticoagulation or liver‐function monitoring [31]. In this study, PT was longer in IgG4‐RD than in DLBCL (11.61 ± 1.35 vs. 10.91 ± 0.86 s), indicating possible discriminatory value. Direct evidence linking PT specifically to the distinction between these two disorders is lacking, although DLBCL is known to alter coagulation homeostasis and to carry a clinically relevant risk of venous thromboembolism [32]. Tumor‐associated tissue factor and inflammatory signaling may promote coagulation activation in lymphoma, so the shorter PT observed in the DLBCL group could partly reflect malignancy‐related hemostatic changes; this interpretation remains speculative. Coagulation abnormalities in IgG4‐RD are less well defined. Hepatobiliary involvement may influence coagulation tests, and IgG4 autoantibodies against complement factor H have been described in complement‐mediated thrombotic microangiopathy [33]. These observations offer possible explanations for the PT difference but do not establish a mechanism. Because numerous laboratory variables were screened without formal correction for multiple testing, a chance association is also possible. Nevertheless, PT remained significant in the multivariable model and in the matched biopsy‐proven sensitivity analysis. It should therefore be regarded as an exploratory candidate predictor rather than a disease‐specific biomarker, with confirmation required in larger independent datasets. The direction of the PT difference should also be interpreted carefully because the absolute between‐group difference was modest and PT can be affected by liver function, nutritional status, medications, and other clinical conditions. Our analysis does not establish that either disease directly causes the observed change in PT. Rather, the finding identifies PT as a statistically reproducible signal within this cohort that warrants independent investigation. Future studies with larger samples and more detailed coagulation measurements will be needed to determine whether the association is stable and to clarify its biological basis.

Within the present cohort, the model showed good discrimination (AUC = 0.91) and acceptable calibration, with an internally assessed mean absolute error (MAE) of 0.032. The predicted probabilities were therefore reasonably consistent with observed outcomes in this dataset, although the small sample may lead to optimistic performance estimates. DCA also indicated possible net benefit over “treat‐all” and “treat‐none” strategies across a range of thresholds. These results remain exploratory and should not be interpreted as proof of established clinical utility. The nomogram is intended to complement, not replace, pathological biopsy, which remains the diagnostic gold standard, and may be most useful during initial assessment when IgG4‐RD and DLBCL have overlapping features and tissue confirmation is not immediately available. In particular, the favorable AUC obtained in a small development cohort may overstate performance that would be observed in new patients. For that reason, no conclusion regarding routine implementation can be made from the present internal assessment alone.

The matched biopsy‐proven sensitivity analysis was designed to examine whether diagnostic misclassification could materially affect the main results. After restricting the analysis to pathologically confirmed cases and matching 1:1 for age and sex, PT continued to differ significantly between IgG4‐RD and DLBCL, whereas several other variables showed changes in the same direction without reaching statistical significance. CD10 expression also differed among the immunohistochemical markers available; however, tissue results were limited to a small subset, so this observation should be interpreted cautiously and does not establish causation or a specific mechanism. Persistence of the PT difference suggests that it was not driven solely by clinically diagnosed cases. Even so, the small sample and limited power mean that this subgroup analysis should be considered only a sensitivity analysis, not an independent validation of the nomogram or evidence of causality. Similar directional changes in several variables provide some reassurance that the overall between‐group pattern was not completely dependent on the broader clinical cohort. However, the reduced number of patients means that absence of statistical significance for other predictors may simply reflect limited power.

## 5 Limitations

Several limitations should be considered. The post‐hoc analysis based on PT estimated statistical power at 53.8%, reflecting the constraints of the modest sample. Although the rarity of IgG4‐RD makes recruitment of a large single‐center cohort difficult, the small sample remains an important weakness. Numerous laboratory variables were also explored without formal correction for multiple comparisons, so some statistically significant findings may have occurred by chance; in particular, the association involving PT requires independent confirmation. Pathological confirmation remains central to definitive diagnosis of both IgG4‐RD and DLBCL, and the model should be viewed only as an aid to the diagnostic process. The retrospective, single‐center design and total sample of 56 patients also limit generalizability and increase the possibility of overfitting and optimistic estimates of performance. Although internal analyses suggested useful discrimination and calibration, external validation in larger independent multicenter cohorts is necessary before clinical implementation. In addition, available baseline data did not allow incorporation of certain clinical features, including patterns of organ involvement, or disease‐specific markers such as serum IgG4 and the IgG4/IgG ratio. Future studies should determine whether adding these variables improves discrimination and clinical usefulness. Finally, the biopsy‐proven sensitivity subgroup included only 24 patients, limiting statistical power and preventing multivariable adjustment; its findings should therefore remain exploratory and hypothesis‐generating. These limitations are especially relevant to interpretation of the nomogram, because model development and performance assessment were conducted in the same relatively small dataset. The reported estimates should therefore be regarded as descriptive of this cohort rather than definitive measures of performance in future patients. More complete prospective data collection would also allow standardized assessment of disease activity, treatment exposure, and organ‐specific manifestations.

## 6 Conclusion

In summary, lymphocyte count, serum total protein, and PT emerged as potential variables for distinguishing IgG4‐RD from DLBCL and were combined in an exploratory prediction model. The model showed promising discrimination in this dataset and may offer supplementary information during early diagnostic assessment, while pathological confirmation remains essential. Larger independent studies are needed to validate these results before the model can be considered for broader clinical application, particularly given the limited sample and retrospective single‐center design.

## Author Contributions

**Yongkang Chen:** conceptualization, methodology, investigation, data curation, formal analysis, writing – original draft, visualization. **Weimiao Li:** conceptualization, methodology, validation, formal analysis, writing – review and editing. **Zhou Zhou:** investigation, data curation, visualization. **Jiqing Wu:** investigation, resources, writing – review and editing. **Dongmei Zhou:** writing – review and editing, supervision, funding acquisition.

## Ethics Statement

Written informed consent for all data was obtained from patients during their hospitalization, and the Ethics Committee of the Affiliated Hospital of Xuzhou Medical University approved the study (XYFY2025‐KL206‐01).

## Conflicts of Interest

The authors declare no conflicts of interest.

## Supporting information


Supporting File 1

Supporting File 2

Supporting File 3

## Data Availability

The data that support the findings of this study are available from the corresponding author upon reasonable request.
