# Supplementary material for: Identification of Discriminatory Factors and Construction of a Nomogram for Differentiating IgG4‐RD and DLBCL
Source: Immun Inflamm Dis. 2026 Sep 28;14(9):e70529. doi: 10.1002/iid3.70529 (PMC13618381; doi:10.1002/iid3.70529)
Supplement: Supplementary file 2 [file IID3-14-e70529-s002.pdf]

STROBE Statement—checklist of items that should be included in reports of observational studies

	Item No.	Recommendation	Page No.	Relevant text from manuscript
**Title and abstract**	1	(*a*) Indicate the study's design with a commonly used term in the title or the abstract	1-2	The title indicates the study involves differentiation of IgG4-RD and DLBCL; abstract states data from 19 patients initially diagnosed with IgG4-RD and 37 patients initially diagnosed with DLBCL (presenting with lymphadenopathy) were retrospectively analyzed.
		(*b*) Provide in the abstract an informative and balanced summary of what was done and what was found	2	Structured summary includes Objective, Methods, Results, and Conclusion; key findings (AUC = 0.91, 95% CI: 0.82–0.99) and independent predictors (lymphocyte count, serum total protein, PT) are reported
**Introduction**				
Background/rationale	2	Explain the scientific background and rationale for the investigation being reported	3-4	Explains the clinical overlap between IgG4-RD and DLBCL, diagnostic challenges, limitations of current diagnostic approaches, and the need for a non-invasive predictive model
Objectives	3	State specific objectives, including any prespecified hypotheses	4-5	To identify discriminatory indicators via retrospective analysis and develop a non-

				invasive predictive model to facilitate early differential diagnosis of IgG4-RD and DLBCL
**Methods**				
Study design	4	Present key elements of study design early in the paper	5	19 patients initially diagnosed with IgG4-RD and 37 patients initially diagnosed with DLBCL (presenting with lymphadenopathy) were retrospectively enrolled
Setting	5	Describe the setting, locations, and relevant dates, including periods of recruitment, exposure, follow-up, and data collection	5	retrospectively enrolled at the Affiliated Hospital of Xuzhou Medical University between January 2012 and October 2025
Participants	6	(*a*) *Cohort study*—Give the eligibility criteria, and the sources and methods of selection of participants. Describe methods of follow-up<br>*Case-control study*—Give the eligibility criteria, and the sources and methods of case ascertainment and control selection. Give the rationale for the choice of cases and controls<br>*Cross-sectional study*—Give the eligibility criteria, and the sources and methods of selection of participants	5-6	Inclusion and exclusion criteria for IgG4-RD and DLBCL groups are listed in Methods → Study Population. IgG4-RD diagnosed by ACR/EULAR 2019 or Japanese 2011 criteria; DLBCL by NCCN/ESMO criteria.
		(*b*) *Cohort study*—For matched studies, give matching criteria and number of exposed and unexposed<br>*Case-control study*—For matched studies, give matching criteria and the number of controls per case	6	1:1 greedy nearest-neighbor matching by age (±10 years) and gender performed in the biopsy-proven subgroup; 12 pairs (12 IgG4-RD, 12 DLBCL) generated; age and gender balanced (P = 0.754 and P = 1.000, respectively)

Variables	7	Clearly define all outcomes, exposures, predictors, potential confounders, and effect modifiers. Give diagnostic criteria, if applicable	6	A total of 62 parameters were collected within 24 hours of hospital admission, encompassing laboratory indicators (e.g., complete blood count, biochemical tests, selected immunological assays) and demographic data (e.g., age, sex, body mass index, disease duration)
Data sources/ measurement	8*	For each variable of interest, give sources of data and details of methods of assessment (measurement). Describe comparability of assessment methods if there is more than one group	6	All parameters were collected within 24 hours of hospital admission; laboratory assays were performed using standard clinical laboratory protocols at the Affiliated Hospital of Xuzhou Medical University
Bias	9	Describe any efforts to address potential sources of bias	6-7	Sensitivity analysis in biopsy-proven subgroup with 1:1 matching by age and gender; immunohistochemical evaluation performed to validate pathological consistency; consistent effect directions across full cohort and matched subgroup
Study size	10	Explain how the study size was arrived at	23-24	post-hoc power analysis based on the most credible predictor PT yielded a power of 53.8%, reflecting the limited sample size inherent to the rarity of

	IgG4-RD; sample size was determined by case availability, not statistical design

Continued on next page

Quantitative variables	11	Explain how quantitative variables were handled in the analyses. If applicable, describe which groupings were chosen and why	6-7	Normality assessed via Shapiro-Wilk test; normally distributed variables presented as mean ± SD and compared by independent t-test; non-normally distributed variables presented as median (IQR) and compared by Mann-Whitney U test
Statistical methods	12	(*a*) Describe all statistical methods, including those used to control for confounding	6-7	Independent t-test, Mann-Whitney U test, chi-square test, multivariate logistic regression, ROC curves, calibration curves, decision curve analysis (DCA); all described in detail
		(*b*) Describe any methods used to examine subgroups and interactions	Not applicable	No subgroup or interaction analyses were performed
		(*c*) Explain how missing data were addressed	6-7	Missing data were not imputed, and analyses were conducted on available cases only
		(*d*) *Cohort study*—If applicable, explain how loss to follow-up was addressed<br>*Case-control study*—If applicable, explain how matching of cases and controls was addressed<br>*Cross-sectional study*—If applicable, describe analytical methods taking account of sampling strategy	7	1:1 greedy nearest-neighbor matching by age (±10 years) and gender described in Methods → Statistical Analysis
		(*e*) Describe any sensitivity analyses	6-7	Sensitivity analysis described; Sensitivity analysis in matched biopsy-proven subgroup reported in Table 2
**Results**				
Participants	13*	(a) Report numbers of individuals at each stage of study—eg numbers potentially eligible, examined for eligibility, confirmed eligible, included in the study, completing follow-up, and analysed	7-8,13	56 patients, comprising 19 in the IgG4-RD group and 37 in the DLBCL group, 12 pairs (24

				patients) after matching
		(b) Give reasons for non-participation at each stage	Not applicable	This is a retrospective study; no participants were lost to follow-up or excluded after enrollment. The exclusion criteria were applied prior to analysis
		(c) Consider use of a flow diagram	Not included	A flow diagram was not used; however, the study population and matched subgroup are clearly described in the text and tables (Table 1 and Table 2)
Descriptive data	14*	(a) Give characteristics of study participants (eg demographic, clinical, social) and information on exposures and potential confounders	7-20	Baseline demographic and laboratory characteristics are presented for both the full cohort and the matched biopsy-proven subgroup
		(b) Indicate number of participants with missing data for each variable of interest	20	Data not available for all patients.
		(c) *Cohort study*—Summarise follow-up time (eg, average and total amount)		
Outcome data	15*	*Cohort study*—Report numbers of outcome events or summary measures over time		
		Case-control study—Report numbers in each exposure category, or summary measures of exposure	20-22	Univariate and multivariate logistic regression estimates (OR, 95% CI, *P* values) for lymphocyte count, serum total protein, PT, eosinophil percentage, and HCO_3^-; , sensitivity, specificity, PPV, NPV with 95% CIs
		Cross-sectional study—Report numbers of outcome events or summary measures		
Main results	16	(*a*) Give unadjusted estimates and, if applicable, confounder-adjusted estimates and their precision (eg, 95% confidence interval). Make clear which confounders were adjusted for and why they were included	20-22	Both unadjusted (univariate) and adjusted (multivariate) ORs and 95% CIs are presented; adjusted variables (lymphocyte count, serum

			total protein, PT) are clearly indicated
	(*b*) Report category boundaries when continuous variables were categorized	Not applicable	The nomogram uses continuous variables (lymphocyte count, serum total protein, PT) without categorizing them; the cut-off value (0.354) is provided only for the ROC curve in Table 4
	(*c*) If relevant, consider translating estimates of relative risk into absolute risk for a meaningful time period	Not performed	Not applicable for this diagnostic prediction model study

Continued on next page

Other analyses	17	Report other analyses done—eg analyses of subgroups and interactions, and sensitivity analyses	13-20,23-24	Sensitivity analysis in matched biopsy-proven subgroup (Table 2) and post-hoc power analysis reported in Results
**Discussion**				
Key results	18	Summarise key results with reference to study objectives	24-26	Key results: lymphocyte count, serum total protein, and PT were independent predictors; nomogram demonstrated good discriminative ability (AUC = 0.91, 95% CI: 0.82–0.99) and calibration (MAE = 0.032)
Limitations	19	Discuss limitations of the study, taking into account sources of potential bias or imprecision. Discuss both direction and magnitude of any potential bias	29-31	Power = 53.8% acknowledged; small sample size (n=56); single-center, retrospective design; model may be susceptible to overfitting; IHC data incomplete; external validation needed
Interpretation	20	Give a cautious overall interpretation of results considering objectives, limitations, multiplicity of analyses, results from similar studies, and other relevant evidence	29-31	Cautious interpretation provided; nomogram positioned as screening tool, not replacement for biopsy; calls for external validation; findings considered preliminary and hypothesis-generating
Generalisability	21	Discuss the generalisability (external validity) of the study results	29-31	single-center, retrospective design and relatively small sample size (n = 56) limit the generalizability of the findings; external validation in multi-center, prospective cohorts needed
**Other information**				

Funding	22	Give the source of funding and the role of the funders for the present study and, if applicable, for the original study on which the present article is based	38	This study was supported by LCZX202413, the funder had no role in study design, data collection, analysis, or manuscript preparation.

*Give information separately for cases and controls in case-control studies and, if applicable, for exposed and unexposed groups in cohort and cross-sectional studies.

Note: An Explanation and Elaboration article discusses each checklist item and gives methodological background and published examples of transparent reporting. The STROBE checklist is best used in conjunction with this article (freely available on the Web sites of PLoS Medicine at http://www.plosmedicine.org/, Annals of Internal Medicine at http://www.annals.org/, and Epidemiology at http://www.epidem.com/). Information on the STROBE Initiative is available at www.strobe-statement.org.
