# Supplementary material for: Identification of Discriminatory Factors and Construction of a Nomogram for Differentiating IgG4‐RD and DLBCL
Source: Immun Inflamm Dis. 2026 Sep 28;14(9):e70529. doi: 10.1002/iid3.70529 (PMC13618381; doi:10.1002/iid3.70529)
Supplement: Supplementary file 3 [file IID3-14-e70529-s003.docx]

**Supplementary Table S1 The 2019 American College of Rheumatology/European League Against Rheumatism classification criteria**

| **The 2019 American College of Rheumatology/European League Against Rheumatism classification criteria for IgG_4_-RD** | |
| --- | --- |
| **Step** | **Categorical assessment or numerical weight** |
| **Step 1. Entry criteria** | |
| Characteristic* clinical or radiological involvement of a typical organ (eg, pancreas, salivary glands, bile ducts, orbits, kidney, lung,  aorta, retroperitoneum, pachymeninges or thyroid gland  (Riedel’s thyroiditis)) OR pathological evidence of an inflammatory process accompanied by a lymphoplasmacytic infiltrate of uncertain aetiology in one of these same organs | Yes† or No |
| **Step 2. Exclusion criteria: domains and items‡** | **Yes or No§** |
| **Clinical** | |
| Fever | |
| No objective response to glucocorticoids | |
| **Serological** | |
| Leucopenia and thrombocytopenia with no explanation | |
| Peripheral eosinophilia | |
| Positive antineutrophil cytoplasmic antibody (specifically against proteinase 3 or myeloperoxidase) | |
| Positive SSA/Ro or SSB/La antibody | |
| Positive double-stranded DNA, RNP or Sm antibody | |
| Other disease-specific autoantibody | |
| Cryoglobulinemia | |
| **Radiological** | |
| Known radiological findings suspicious for malignancy or infection that have not been sufficiently investigated | |
| Rapid radiological progression | |
| Long bone abnormalities consistent with Erdheim-Chester disease | |
| Splenomegaly | |
| **Pathological** | |
| Cellular infiltrates suggesting malignancy that have not been sufficiently evaluated | |
| Markers consistent with inflammatory myofibroblastic tumour | |
| Prominent neutrophilic inflammation | |
| Necrotizing vasculitis | |
| Prominent necrosis | |
| Primarily granulomatous inflammation | |
| Pathologic features of macrophage/histiocytic disorder | |
| **Known diagnosis of the following:** | |
| Multicentric Castleman’s disease | |
| Crohn’s disease or ulcerative colitis (if only pancreatobiliary disease is present) | |
| Hashimoto thyroiditis (if only the thyroid is affected) | |
| **If case meets entry criteria and does not meet any exclusion criteria, proceed to step 3.** | |
| **Step 3. Inclusion criteria: domains and items¶** | |
| **Histopathology** | |
| Uninformative biopsy | 0 |
| Dense lymphocytic infiltrate | +4 |
| Dense lymphocytic infiltrate and obliterative phlebitis | +6 |
| Dense lymphocytic infiltrate and storiform fibrosis with or without obliterative phlebitis +13 | |
| **Immunostaining**** | **0–16, as follows:** |
| Assigned weight is 0 if the IgG_4_+:IgG+ ratio is 0%–40% or indeterminate and the number of IgG_4_+ cells/hpf is 0–9.‡‡  Assigned weight is 7 if: (1) the IgG_4_+:IgG+ ratio is ≥41% and the number of IgG_4_+cells/hpf is 0–9 or indeterminate or (2) the IgG_4_+:IgG+ ratio is 0–40% or indeterminate and the number of IgG_4_+ cells/hpf is ≥10 or indeterminate.  Assigned weight is 14 if: (1) the IgG_4_+:IgG+ ratio is 41%–70% and the number of IgG_4_+ cells/hpf is ≥10 or (2) the IgG_4_+:IgG+ ratio is ≥71% and the number of IgG_4_+ cells/hpf is 10–50.  Assigned weight is 16 if the IgG_4_+:IgG+ ratio is ≥71% and the number of IgG_4_+ cells/hpf is ≥51. | |
| **Serum IgG_4_ concentration** | |
| Normal or not checked | 0 |
| >Normal but <2× upper limit of normal | +4 |
| 2–5× upper limit of normal | +6 |
| ≥>5× upper limit of normal | +11 |
| **Bilateral lacrimal, parotid, sublingual and submandibular glands** | |
| **Table 4 Continued** | |
| **Step** | **Categorical assessment or numerical weight** |
| No set of glands involved | 0 |
| One set of glands involved | +6 |
| Two or more sets of glands involved | +14 |
| **Chest** | |
| Not checked or neither of the items listed is present | 0 |
| Peribronchovascular and septal thickening | +4 |
| Paravertebral band-like soft tissue in the thorax | +10 |
| **Pancreas and biliary tree** | |
| Not checked or none of the items listed is present | 0 |
| Diffuse pancreas enlargement (loss of lobulations) | +8 |
| Diffuse pancreas enlargement and capsule-like rim with decreased enhancement | +11 |
| Pancreas (either of above) and biliary tree involvement | +19 |
| **Kidney** | |
| Not checked or none of the items listed is present | 0 |
| Hypocomplementemia | +6 |
| Renal pelvis thickening/soft tissue | +8 |
| Bilateral renal cortex low-density areas | +10 |
| **Retroperitoneum** | |
| Not checked or neither of the items listed is present | 0 |
| Diffuse thickening of the abdominal aortic wall | +4 |
| Circumferential or anterolateral soft tissue around the infrarenal aorta or iliac arteries | +8 |
| **Step 4: Total inclusion points** | |
| A case meets the classification criteria for IgG_4_-RD if the entry criteria are met, no exclusion criteria are present, and the total  points is ≥20 | |

*Refers to enlargement or tumour-like mass in an affected organ except in (1) the bile ducts, where narrowing tends to occur, (2) the aorta, where wall thickening or aneurysmal dilatation is typical and (3) the lungs, where thickening of the bronchovascular bundles is common.

†If entry criteria are not fulfilled, the patient cannot be further considered for classification as having IgG_4_-RD.

‡Assessment for the presence of exclusion criteria should be individualised depending on a patient’s clinical scenario.

§If exclusion criteria are met, the patient cannot be further considered for classification as having IgG_4_-RD.

¶Only the highest weighted item in each domain is scored.

**Biopsies from lymph nodes, mucosal surfaces of the gastrointestinal tract and skin are not acceptable for use in weighting the immunostaining domain.

††‘Indeterminate’ refers to a situation in which the pathologist is unable to clearly quantify the number of positively staining cells within an infiltrate, yet can still ascertain that the number of cells is at least 10/hpf. For a number of reasons, most often pertaining to the quality of the immunostain, pathologists are sometimes unable to count the number of IgG_4_+ plasma cells with precision yet even so, can be confident in grouping cases into the appropriate immunostaining result category.

hpf, high-power field; IgG_4_-RD, IgG_4_-related disease.

**Supplementary Table S2 The 2011 Japanese comprehensive diagnostic criteria**

| **Comprehensive clinical diagnostic criteria for IgG4-RD** | |
| --- | --- |
| **Section** | **Criteria or description** |
| **I. Concept** | **IgG4-related disease (IgG4-RD) shows organ enlargement or nodular/hyperplastic lesions in various organs concurrently or metachronously, due to marked infiltration of lymphocytes and IgG4? plasma cells, as well as fibrosis of unknown etiology. IgG4-RD affects various organs, including the pancreas, bile duct, lacrimal gland, salivary gland, central nervous system, thyroid, lung, liver, gastrointestinal tract, kidney, prostate, retroperitoneum, arteries, lymph nodes, skin, and breast. Although many patients with IgG4-RD have lesions in several organs, either synchronously or metachronously, others show involvement of a single organ. Clinical symptoms vary depending on the affected organ, and some patients may experience serious complications, such as obstruction or compression symptoms due to organomegaly or hypertrophy, and organ dysfunction caused by cellular infiltration or fibrosis. Steroid therapy is often effective** |
| II. Comprehensive clinical diagnostic criteria for IgG4-RD | |
| 1. Clinical findings | 1. Clinical examination showing characteristic diffuse/localized swelling or masses in single or multiple organs |
| 2. Serological findings | 2. Hematological examination shows elevated serum IgG4 concentrations( 135 mg/dl) |
| 3. Histopathological findings | 3. Histopathologic examination shows: (1) Marked lymphocyte and plasmacyte infiltration and fibrosis. (2) Infiltration of IgG4+ plasma cells: ratio of IgG4+/IgG+ cells > 40% and >10 IgG4+ plasma cells/HPF |
| Diagnostic categories | Definite: 1) + 2) + 3); Probable: 1) + 3); Possible: 1) + 2) |
| Differential diagnosis | However, it is important to differentiate IgG4-RD from malignant tumors of each organ (e.g. cancer, lymphoma) and similar diseases (e.g. Sjögren’s syndrome, primary sclerosing cholangitis, Castleman’s disease, secondary retroperitoneal fibrosis, Wegener’s granulomatosis, sarcoidosis, Churg–Strauss syndrome) by additional histopathological examination |
| **Organ-specific criteria** | **Even when patients cannot be diagnosed using the CCD criteria, they may be diagnosed using organ specific diagnostic criteria for IgG4RD** |
| III. Explanatory notes | |
| 1. General application | 1. The comprehensive diagnostic criteria are the minimal consensus to aid general practitioners and other nonspecialist physicians in the clinical diagnosis of IgG4-RD. For each affected organ, organ-specific diagnostic criteria established for IgG4-related Mikulicz’s disease, IgG4-related autoimmune pancreatitis, and IgG4-related kidney disease, should be used concurrently |
| 2. Concept | The difference from multifocal fibrosclerosis is unclear although these diseases may be IgG4-RD. Many patients show multiple organ involvement and are characterized as having systemic disease, whereas other patients show involvement of a single organ |
| (a) | Autoimmune pancreatitis, type 1 (IgG4-related autoimmune pancreatitis): This disease is synonymous with IgG4-related sclerosing pancreatitis/lymphoplasmacytic sclerosing pancreatitis (LPSP). It can be diagnosed using the clinical diagnostic criteria for autoimmune pancreatitis established by the Ministry of Health, Labor and Welfare, Japan Pancreas Society, in 2006 . |
| (b) | IgG4-related sclerosing cholangitis: This disease is characterized by sclerotic changes with diffuse or localized stenosis in the intrahepatic/extrahepatic bile duct and gallbladder. Circumferential wall thickening is observed at the site of stenosis, with similar changes in areas without stenosis. Obstructive jaundice often develops, making it important to differentiate this condition from tumors, such as cholangiocarcinoma and pancreatic cancer, and from primary sclerosing cholangitis. It is also necessary to exclude secondary sclerosing cholangitis as an apparent cause |
| (c) | IgG4-related lacrimal, orbital, and salivary-gland lesions: This condition includes IgG4-related Mikulicz’s disease characterized by symmetrical (sometimes unilateral) swelling of any of the lacrimal, parotid, submandibular, sublingual glands, and some minor salivary glands. Nodular/infiltrative lesions may also occur in orbital tissue other than the lacrimal glands. IgG4-related Mikulicz’s disease can be diagnosed by the organ-specific diagnostic criteria for IgG4-related Mikulicz’s disease established by the Sjogren’s Syndrome Study Group of Japan in 2008 |
| (d) | IgG4-related central nervous system lesions: These lesions include infundibular hypophysitis, hypertrophic pachymeningitis, and intracerebral inflammatory pseudotumor |
| (e) | IgG4-related respiratory lesions: These lesions occur primarily in the interstitium, such as bronchovascular bundles, interlobular septum, alveolar septum, and pleura. They are frequently accompanied by mediastinal and hilar lymphadenopathy, along with X-ray evidence of a mass or infiltration of the lung. Some patients have asthma-like symptoms. It is important to differentiate these lesions from malignant tumors, sarcoidosis, collagen diseases of the lung, and infection |
| (f) | IgG4-related renal lesions: Abnormal imaging findings include diffuse renal enlargement, multifocal contrast defects of the renal parenchyma, renal mass lesions, and pelvic wall thickening. Renal histology shows mainly interstitial nephritis, but glomerular lesions (e.g., membranous nephropathy), may also be present. IgG4-related tubulointerstitial nephritis can be diagnosed using the organ-specific diagnostic criteria for IgG4-related kidney disease |
| (g) | IgG4-related retroperitoneal fibrosis/periarterial lesions: This disease is characterized by thickening of the abdominal aortic adventitia and periurethral soft tissue, often accompanied by hydronephrosis or mass lesions. Periarteritis may occur around the aorta or relatively large branches and is evident as arterial wall thickening on radiological imaging. Magnetic resonance imaging (MRI) and positron emission tomography (PET) have been shown to be helpful for diagnosing retroperitoneal fibrosis in addition to X-ray, which may include CT scan. |
|  | Biopsy is often inconclusive, making it difficult to differentiate this condition from secondary retroperitoneal fibrosis due to malignant tumors or infectious diseases |
| **(h)** | **Other tumefactive lesions: Proliferation of IgG4? plasma cells and lymphocytes may accompany fibrosis. Including some** |
|  | conventional inflammatory pseudotumors, these lesions have been reported in the brain, orbit, lung, breast, liver, pancreas, retroperitoneum, kidney, and lymph nodes |
| IV. Blood test findings | |
| 1. | Polyclonal serum c-globulin, IgG, and IgE are often elevated, and hypocomplementemia may occur |
| 2. | Elevated IgG4 can also be seen in other diseases (e.g., atopic dermatitis, pemphigus, asthma, and multicentric Castleman’s disease) and is therefore not specific to IgG4-RD |
| 3. | On rare occasions, serum IgG4 concentration may be elevated in patients with malignant tumors. However, patients with [270 mg/dl IgG4 are unlikely to have pancreatic cancer |
| **4.** | **In patients with single-organ involvement and serum IgG4 concentration\135 mg/dl, the IgG4?/IgG? ratio may be helpful in making a diagnosis** |
| 5. | At present, the significance of elevated IgG4 in the pathogenesis/pathophysiology of IgG4-RD is unknown |
| V. Histopathological findings | |
| 1. | Storiform or swirling fibrosis or obliterative phlebitis are characteristic of IgG4-RD and may be important in its diagnosis |
| **2.** | **Eosinophilic infiltration often occurs, along with infiltration of IgG4? cells** |
| 3. | Reactive infiltration of IgG4? cells and fibrosis may also occur, such as at the periphery of pancreatic cancers |
| VI. Imaging studies | IgG4-RD may occur, either synchronously or metachronously, in a variety of organs throughout the body, including the pancreas, bile duct, lacrimal gland, salivary gland, thyroid, lung, liver, gastrointestinal tract, kidney, and retroperitoneum. MRI and fluorodeoxyglucose (FDG)- PET have been shown to be helpful for detecting multiorgan involvements |
| VII. Steroids | |
| 1. | Patients with malignant lymphoma or paraneoplastic lesions can sometimes be improved by steroid administration. Therefore, steroid trials should be strictly avoided |
| 2. | Efforts should be made to collect tissue samples for diagnosis. However, patients having disease in organs difficult to biopsy, such as pancreas, retroperitoneum, and pituitary, and who respond to steroids may possibly have IgG4-RD |
| 3. | In accordance with the guidelines for treatment of autoimmune pancreatitis, patients should be started on 0.5–0.6 mg/kg per day of prednisolone. If patients do not respond to the initial steroid therapy, the diagnosis should be reviewed |
| VIII. Diseases to be excluded or differentiated | |
| 1. | To exclude malignancies (e.g., cancer, lymphoma) in involved organs, it is essential to determine histopathologically whether malignant cells are present |
| 2. | Similar diseases (e.g., Sjo¨gren’s syndrome, primary sclerosing cholangitis, multicentric Castleman’s disease, idiopathic retroperitoneal fibrosis, Wegener’s granulomatosis, sarcoidosis, Churg–Strauss syndrome) are diagnosed using the diagnostic criteria for each disease |
| 3. | Multicentric Castleman’s disease is a hyper-interleukin (IL)-6 syndrome and is not included among the IgG4-RDs, even if the diagnostic criteria for IgG4-RD are fulfilled |
